# Microwave Synthesis of Gold Nanoclusters with Garlic Extract Modifications for the Simple and Sensitive Detection of Lead Ions

**DOI:** 10.3390/nano10010094

**Published:** 2020-01-02

**Authors:** Lingaraj Ryavanaki, Hweiyan Tsai, C. Bor Fuh

**Affiliations:** 1 Department of Applied Chemistry, National Chi Nan University, Nantou 545, Taiwan; s103324903@mail1.ncnu.edu.tw; 2 Department of Medical Applied Chemistry, Chung Shan Medical University, Taichung 402, Taiwan; 3 Department of Medical Education, Chung Shan Medical University Hospital, Taichung 402, Taiwan

**Keywords:** BSA-gold nanoclusters (BSA-AuNCs), garlic extract, lead ions

## Abstract

Novel bovine serum albumin (BSA)-gold nanoclusters with garlic extract modifications (*mw*_G-BSA-AuNCs) were prepared through microwave-assisted rapid synthesis. The modified nanoclusters were characterized and used for the simple and sensitive detection of Pb^2+^ ions. Both turn-on and turn-off methods were used and compared for Pb^2+^ ion detection. For Pb^2+^ ions, the preparation time for the modified nanoclusters was 10 min, and the detection time for the nanoclusters was 6 min. The modified nanoclusters were stable, and their fluorescence intensities changed by less than 10% in 60 days. The detection limit and linear range for the “off-on” method of *mw*_G-BSA-AuNCs for Pb^2+^ ion detection were 0.28 and 1–20 nM, respectively. The recoveries of the *mw*_G-BSA-AuNCs probe used for the detection of the Pb(II) ion in tap water ranged from 93.8% to 102.2%, with an average of 97.1%. The “off-on” method of *mw*_G-BSA-AuNCs can provide a lower detection limit, higher selectivity, and better recovery than the commonly used “turn-off” methods of *mw*_BSA-AuNCs for Pb^2+^ ion detection. The proposed method is superior to other methods proposed from 2018 to 2019 because it can provide a shorter preparation time and a lower detection limit with good selectivity. The microwave-assisted novel compound, *mw*_G-BSA-AuNCs, can be rapidly synthesized in a green manner and can provide a low detection limit, good selectivity, and a simple and fast reaction for Pb^2+^ ion detection.

## 1. Introduction

Lead [Pb(II), Pb^2+^] ions are known to be heavy metals and environmental pollutants. They can cause serious threats to human health and ecological environments. Lead ions are problematic because they are water-soluble, nonbiodegradable, long-lasting, and easily accumulate in the human body. Lead ions can enter the body through air, food, soil, and water. Low concentrations of Pb^2+^ ions can cause various adverse and even toxic effects to the human body. The Environmental Protection Agency of the United States and the World Health Organization have recommended 15 and 10 µg L^−1^ (ppb), respectively, as toxic concentrations of Pb^2+^ ions in drinking water [1,2]. Therefore, sensitive and selective detection methods for Pb^2+^ ions are essential in monitoring, removing, and preventing hazardous effects on living systems. Modern analytical methods such as inductively coupled plasma-mass spectrometry (ICPMS), X-ray fluorescent spectrometry, and atomic absorption and emission spectrometry have been used for the trace detection of heavy metals. However, the instruments are expensive, experienced analysts are required, and the procedures of sample treatment are time-consuming. Therefore, alternative simple, rapid, sensitive, and selective analytical methods must be developed for the detection of Pb^2+^ ions.

Various nanomaterials have been used for heavy metal detection to enhance sensitivity, selectivity, and reproducibility with high surface reactivity, size-dependent properties, and thermal stability [3,4,5,6,7]. Methods based on fluorescent nanomaterials are still favored for applications of heavy metal detection due to their high sensitivities. Semiconductor quantum dots (QDs) are a good alternative to dye-based fluorescent nanomaterials because of their broad absorption spectra, good quantum yields, narrow emission spectra, and high resistance to photobleaching. However, some heavy metal components of QDs have been investigated for potential toxic effects in applications. Gold nanoclusters (AuNCs) have become popular for fluorescent probe applications because of their ultra-small size, easy preparation, and good photostability [8,9,10,11]. Bovine serum albumin (BSA)-modified AuNCs (BSA-AuNCs) have gained attention and popularity in applications of aqueous solutions due to their excellent biocompatibility and abundant functional groups [8,9,10]. Most BSA-AuNC preparations require at least several hours, even if the preparation methods are matured. These preparations are commonly used for metal ion detection in methods of quenching (turning off) fluorescence [9,12]. Developing alternative methods for the rapid and green preparation of BSA-AuNCs is essential for sensitive heavy metal detection in analytical applications.

Extracts of allium vegetables (e.g., onions and garlic) can act as reducing and stabilizing agents for green preparation methods of nanoparticle formation [13,14,15,16,17]. Microwave heating can provide uniform heating and rapid reactions for synthesis applications. Therefore, we summarized all these facts and developed a new strategy of rapid microwave-assisted preparation of garlic extract (GE)-modified BSA-AuNCs (*mw*_G-BSA-AuNCs) for simple, rapid, sensitive, and selective Pb^2+^ ion detection. This rapid and green method provides simple, sensitive, and selective detection through the strategy of “turn-off” and then “turn-on” (off-on) fluorescence methods. We also used BSA-gold nanoclusters (*mw*_BSA-AuNCs) with the commonly used “turn-off” (fluorescence quenching) method as a reference for comparison.

## 2. Experimental Section

### 2.1. Materials and Instruments

Hydrogen tetrachloroaurate(III) trihydrate (HAuCl_4_·3H_2_O), sodium hydroxide, bovine serum albumin, and phosphate buffer saline (PBS) were purchased from Sigma-Aldrich (St. Louis, MO, USA). Fresh garlic cloves were obtained from a local market in Puli, Nantou, Taiwan.

A fluorescence spectrometer (Lumina, Thermo Fisher, Waltham, MA, USA), an infrared spectrometer (Frontier PerkinElmer, Waltham, MA, USA), a UV-Vis spectrometer (Agilent Technologies, Cary 8454, Santa Clara, CA, USA), and a transmission electron microscope (JEOL, JEM-1400, Tokyo, Japan) were used to characterize the materials. Microwave systems were obtained from Milestone (ETHOS-UP, MA182-001, Sorisole, Italy), and centrifuges were purchased from Hettich GmbH (Universal 30RF, Tuttlingen, Germany). 

### 2.2. Preparation of the Aqueous Garlic Extract (GE)

Aqueous GE preparation was conducted as mentioned in the following text. First, garlic cloves were washed three times in ultra-pure deionized water to remove any dust from their outer surfaces. Then, the cloves were ground to a very fine clay-like paste, transferred to 20 mL vials and sonicated for 2 h at room temperature. The sonicated solutions were then centrifuged at 8000 rpm for 10 min, filtered through 0.22 µM membranes, and stored at 4 °C for further use.

### 2.3. Preparation and Optimization of the mw_BSA-AuNC and mw_G-BSA-AuNC Materials 

Both the BSA-AuNCs and G-BSA-AuNCs in the experiment were synthesized with microwave assistance. Before the sample mixtures were microwaved, certain general pretreatments were performed. A total of 0.5 mL of 1.0 M of sodium hydroxide solution was added into mixed aqueous solutions of HAuCl_4_·3H_2_O (10 mM, 2.5 mL) and BSA (50 mg mL^−1^, 2.5 mL) and sonicated for 30 s. Then, 5 mL of deionized water was added into each aforementioned sonicated solution for the preparation of BSA-AuNCs. Deionized water (2.5 mL) and GE [various concentrations (0–222 mg mL^−1^) for optimization, 2.5 mL] were added into the aforementioned samples, which were then sonicated for an additional 30 s for the preparation of G-BSA-AuNCs. The final mixed samples of the sonicated solutions were transferred to microwave Teflon tubes, and the microwave oven was adjusted for different reaction temperatures (50, 60, 70, 80, and 90 °C) and different reaction times (5, 8, 10, 12, 15, and 20 min), according to an optimization process. The products obtained after the microwave reactions were purified through centrifugation in a 3 K centrifugal membrane at a speed of 8000 rpm for 25 min (with an interval time of 5 min). The final products were freeze-dried for further use.

#### 2.3.1. Optimization of *mw*_G-BSA-AuNCs

All the optimization experiments followed the general pretreatments of sample mixtures before the samples were microwaved. After the products had undergone microwave reactions, the products were purified as described previously.

##### Optimization of the Microwave Reaction Temperature

The final mixture samples of sonicated solutions were transferred to microwave Teflon tubes and reacted for 10 min. The microwave reaction temperature was changed from 50 to 90 °C (50, 60, 70, 80, and 90 °C) at 100 W. The product was then purified using the aforementioned purification steps.

##### Optimization of the Microwave Reaction Time

We optimized the synthesis conditions. The final mixtures of sonicated solutions were transferred to microwave Teflon tubes for a microwave reaction at 70 °C (100 W) for different reaction times (5, 8, 10, 12, 15, and 20 min). The obtained products were purified.

##### Optimization of GE Concentrations

The microwave reaction temperature (70 °C) and reaction time (10 min) were maintained constant, and the GE concentrations were changed from 0 to 222 mg mL^−1^. The obtained products were purified.

#### 2.3.2. Preparation of *mw*_G-BSA-AuNCs and *mw*_BSA–AuNCs

The optimal microwave reaction time (10 min), reaction temperature (70 °C), and GE concentration (55 mg mL^−1^) were used in the preparation of *mw*_G-BSA-AuNCs as the general pretreatments of sample mixtures and the purification steps of products. A similar preparation procedure was used for each sample of *mw*_BSA–AuNCs; however, no GE was added, and an additional 2.5 mL of deionized water was added to maintain the same total volume. 

### 2.4. Detection of Pb(II) Ions

Samples of stock Pb(II) solutions at various concentrations (1, 2, 4, 6, 8, 10, 16, 20 nM) were prepared using double distilled water. In a typical test, *mw*_BSA-AuNC or *mw*_G-BSA-AuNC stock solution (1 mg mL^−1^, 0.65 mL), PBS solution (10 mM, PH= 7.4, 50 µL), and metal ion solution samples with various concentrations (100 µL) were incubated for 6 min at room temperature (25 °C). Then, the fluorescence emission intensities were recorded at an excitation wavelength at 370 nm for the detection of metal ions. The same method was applied for the detection of Pb(II) ions by spiking known concentrations of ions into tap water.

## 3. Results and Discussion

### 3.1. Synthesis and Characterization of mw_G-BSA-AuNCs 

BSA not only acts as a stabilizing agent but also as a reductant in the formation of nanoclusters by reducing Au(III) to Au atoms in the form of nanoclusters. Further functionalization was performed by the addition of aqueous GE to the mixture of BSA and Au solutions. GE is rich in organosulfur compounds, cysteine derivatives, polyphenolic compounds, and flavonoid compounds [15,16]. These functional groups of GE caused redox reactions between the functional groups of protein BSA and GE. 

**Figure 1:**
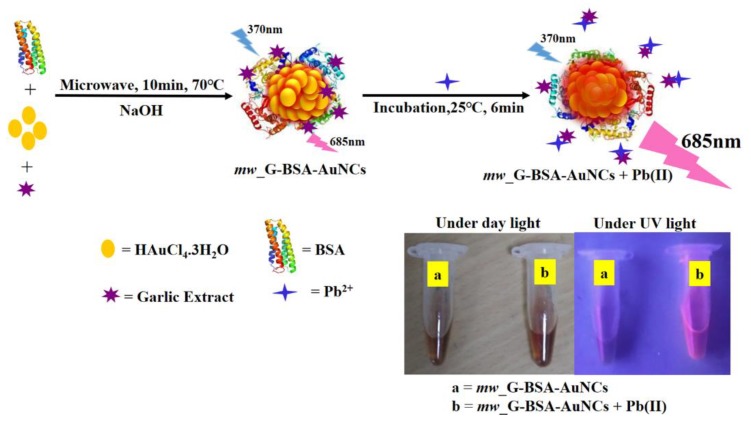
Scheme for the “off-on” method of bovine serum albumin (BSA)-gold nanoclusters with garlic extract modifications (*mw*_G-BSA-AuNCs).

Figure 1 displays the “off-on” strategy of *mw*_G-BSA-AuNCs for Pb^2+^ ion detection. The addition of GE to BSA-AuNCs reduced the fluorescent intensity due to the reactions of AuNCs with the functional groups (organosulfur compounds, polyphenolic compounds, and flavonoid compounds) of GE. When Pb^2+^ ions were added, they strongly reacted with the functional groups of GE and recovered the fluorescence of BSA-AuNCs. Photographs of *mw*_G-BSA-AuNCs (Figure 1a) and *mw*_G-BSA-AuNCs + Pb^2+^ (Figure 1b) under daylight and UV (Ultraviolet) illumination were shot for reference. There was no fluorescence for both compounds under daylight. The fluorescence of *mw*_G-BSA-AuNCs + Pb^2+^ was relatively weak when compared to that of *mw*_G-BSA-AuNCs under UV light. Some quenching methods are commonly used for BSA-AuNCs with Pb^2+^ due to the interactions of Pb^2+^ with the functional groups (amino, carboxylic, and mercapto groups) of BSA.

**Figure 2:**
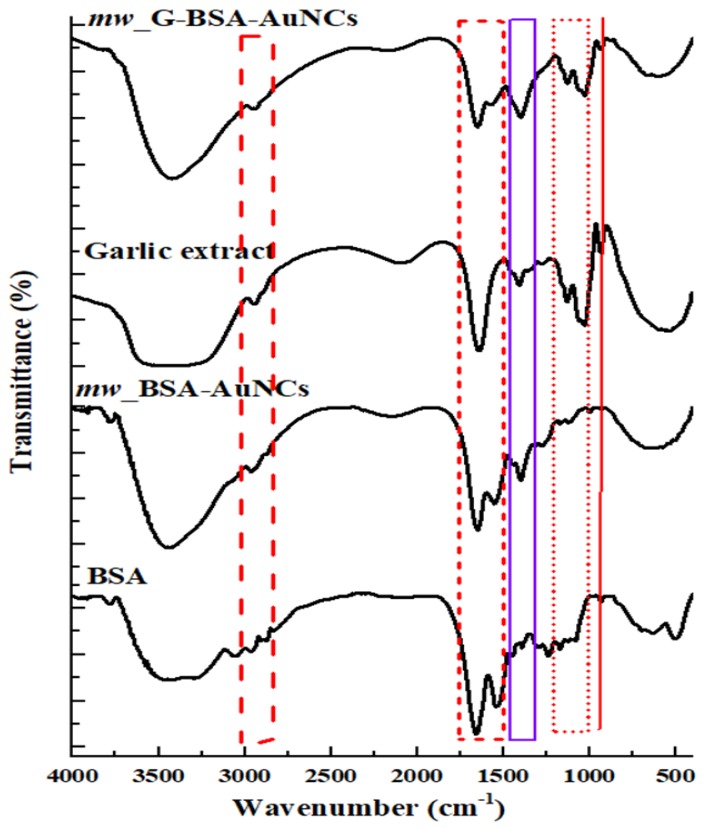
Fourier transform infrared (FTIR) spectra of *mw*_G-BSA-AuNCs, pure garlic extract, BSA-gold nanoclusters (*mw*_BSA-AuNCs) and BSA.

Figure 2 illustrates the infrared spectra of *mw*_BSA-AuNCs, GE, and *mw*_G-BSA-AuNCs obtained through microwave-assisted synthesis. The IR (Infrared) spectra of 3670, 2935, 1664 and 1532 cm^−1^ were assigned to the stretchings associated with the hydroxyl group, C–H bonds, amide–I band, and primary amine scissoring peaks, respectively. The peaks between 1764 and 1235 cm^−1^ were attributed to peptides and protein linkages. The IR spectrum of *mw*_G-BSA-AuNCs exhibited two broad peaks (1139 and 1064 cm^−1^) assigned to the stretching associated with the S=O and C–N bonds in the GE. The peaks at 1424 and 1409 cm^−1^ were assigned to the O–H bending in *mw*_G-BSA-AuNCs and *mw*_BSA-AuNCs, respectively. Figure S1 displays the average particle size distributions for *mw*_G-BSA-AuNCs (2.7 ± 0.5 nm; Figure S1a) and *mw*_BSA-AuNCs (2.5 ± 0.5 nm; Figure S1b). Both the compounds had roughly spherical shapes.

**Figure 3:**
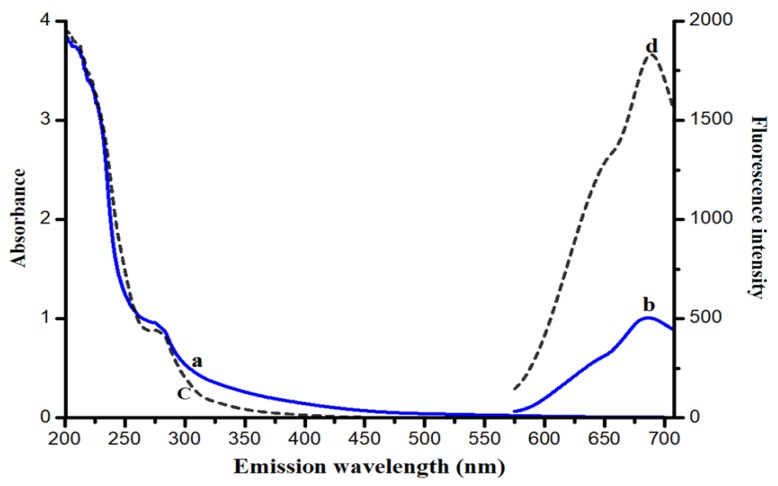
UV-visible and fluorescence emission spectra of *mw*_G-BSA-AuNCs (curves a and b, solid lines) and *mw*_BSA-AuNCs (curves c and d, dashed lines).

Figure 3 illustrates the UV-visible spectra and fluorescence spectra of *mw*_G-BSA-AuNCs (curves a and b, solid lines) and *mw*_BSA-AuNCs (curves c and d, dashed lines). The emission intensity of *mw*_G-BSA-AuNCs was around one fourth of the emission intensity of *mw*_BSA-AuNCs.

#### 3.1.1. Synthesis Optimization of *mw*_G-BSA-AuNCs

Several experimental parameters, including the microwave reaction temperature, reaction time, and GE concentration, were optimized to synthesize *mw*_G-BSA-AuNCs with constant concentrations of BSA (50 mg mL^−1^) and HAuCl_4_·3H_2_O (10 mM). The optimal fluorescent intensity of *mw*_G-BSA-AuNCs should be minimal because the detection concept is based on the “off-on” principle (the detector lights up when it transitions from the “off” state of *mw*_G-BSA-AuNCs to the “on” state of BSA-AuNCs). On the other hand, the optimal fluorescent intensity of *mw*_ BSA-AuNCs should be maximal because the detection concept is based on the “off” principle (quenching the fluorescence intensity of *mw*_ BSA-AuNCs with Pb^2+^ ions).

##### Effect of the Microwave Reaction Temperature on the Fluorescence Intensity of *mw*_G-BSA-AuNCs and *mw*_BSA-AuNCs

The fluorescent intensities of *mw*_G-BSA-AuNCs at different reaction temperatures were measured for a fixed reaction time, at fixed concentrations of GE and HAuCl_4_·3H_2_O. Figure S2a displays the effects of the microwave-induced reaction temperatures on the fluorescence intensity of *mw*_G-BSA-AuNCs at a constant reaction time (10 min) and fixed reactant concentrations (HAuCl_4_·3H_2_O: 10 mM, GE: 55 mg mL^−1^). The emission intensity at a wavelength of 685 nm decreased as the reaction temperature increased from 50 to 70 °C and increased as the reaction temperature increased to 80 and 90 °C for a single fixed reaction time and for several GE concentrations. No shift was observed in the maximal emission wavelength during the synthesis of *mw*_G-BSA-AuNCs with different reaction temperatures. The optimal temperature of the microwave reactions was 70 °C for *mw*_G-BSA-AuNCs.

We also studied the effect of the reaction temperature (from 50 to 80 °C) on the fluorescence of *mw*_BSA-AuNCs as a reference. Some shifts were observed in the emission wavelengths. The emission intensity increased between 50 and 70 °C during the synthesis and decreased at a reaction temperature of 80 °C, as depicted in Figure S3. This decrease in the emission intensity at 80 °C may have been caused by the denaturing effect and aggregation of protein molecules [18,19]. The fluorescence emission of *mw*_BSA-AuNCs at a reaction temperature of 50 °C exhibited two shoulder emission peaks at 645 and 682 nm for an excitation wavelength of 370 nm. This result may have been caused by the incomplete formation of gold nanoclusters at low temperatures. The optimal temperature of the microwave reactions was 70 °C for *mw*_ BSA-AuNCs.

##### Effect of the Reaction Time on the Fluorescence Intensity of *mw*_G-BSA-AuNCs

The variations in the fluorescence intensity of *mw*_G-BSA-AuNCs for different reaction times were determined for a fixed microwave-induced reaction temperature and fixed concentrations of GE and HAuCl_4_·3H_2_O. Figure S2b displays the effects of the microwave reaction time on the fluorescence intensity of *mw*_G-BSA-AuNCs at a constant reaction temperature (70 °C) and fixed reactant concentrations (HAuCl_4_·3H_2_O: 10 mM, GE: 55 mg mL^−1^). The fluorescence emission intensity of *mw*_G-BSA-AuNCs decreased as the reaction time increased from 5 to 10 min at a wavelength of 685 nm with an excitation wavelength of 370 nm. The fluorescence emission intensity increased as the reaction time increased from 10 to 20 min. A reaction time of 10 min was considered to be the optimal value for further study.

##### Effect of the GE Concentration on the Fluorescence Intensity of *mw*_G-BSA-AuNCs

Different concentrations (0 to 222 mg mL^−1^) of GE were added to the reaction mixture while maintaining a constant reaction time (10 min), reaction temperature (70 °C) and fixed reactant concentrations (HAuCl_4_·3H_2_O: 10 mM, BSA: 50 mg mL^−1^) to synthesize *mw*_G-BSA-AuNCs. The fluorescence emission intensity (at 685 nm) of *mw*_G-BSA-AuNCs reached its minimum value when the concentration of GE increased to 55 mg mL^−1^, as displayed in Figure S2c. The optimal GE concentration of 55 mg mL^−1^ was used for further applications.

##### Fluorescence Stability of *mw*_G-BSA-AuNCs Over Different pH Values

Figure S2d illustrates the fluorescence stability of *mw*_G-BSA-AuNCs over different pH values with phosphate buffer (10 mM, pH = 6–12). The fluorescence intensity was lower at acidic and alkaline pH values than at a neutral pH value. A high fluorescence intensity of *mw*_G-BSA-AuNCs was observed within a neutral pH range. 

#### 3.1.2. Characterization of *mw*_G-BSA-AuNCs and *mw*_BSA-AuNCs

The synthesized *mw*_BSA-AuNCs and *mw*_G-BSA-AuNCs were characterized using UV-visible spectroscopy and fluorescence spectroscopy with optimized synthetic conditions. The UV-visible and fluorescence spectra of *mw*_G-BSA-AuNCs and *mw*_BSA-AuNCs are illustrated in Figure 3. We observed no significant band of surface plasmon resonance at 530 nm, which indicated the formation of small nanoclusters instead of large gold nanoparticles. The maximal emission wavelengths were 685 nm for *mw*_G-BSA-AuNCs and 689 nm for *mw*_BSA-AuNCs for the same excitation wavelength of 370 nm. 

##### Fluorescence Stability Over Time of *mw*_G-BSA-AuNCs

The fluorescence stabilities of the as-synthesized *mw*_G-BSA-AuNCs were analyzed for 60 days. The results indicated that the emission intensity of *mw*_G-BSA-AuNCs decreased by less than 10% over 60 days. Thus, the GE-modified BSA-AuNCs had a highly stable fluorescence.

### 3.2. Sensitivity and Selectivity for Pb(II) Detection by mw_G-BSA-AuNCs and mw_BSA-AuNCs Probes

We investigated the effect of the incubation time (1–10 min) during the construction of the sensing platform by using a *mw*_G-BSA-AuNC probe for the detection of 100 nM Pb(II) ions (PBS pH = 7.4, 10 mM). As displayed in Figure S4, the fluorescence intensity ratio (*F*/*F_o_*) of the *mw*_G-BSA-AuNCs dramatically increased as the incubation time reached 6 min, and no further fluorescence enhancement was observed for up to 10 min.

**Figure 4:**
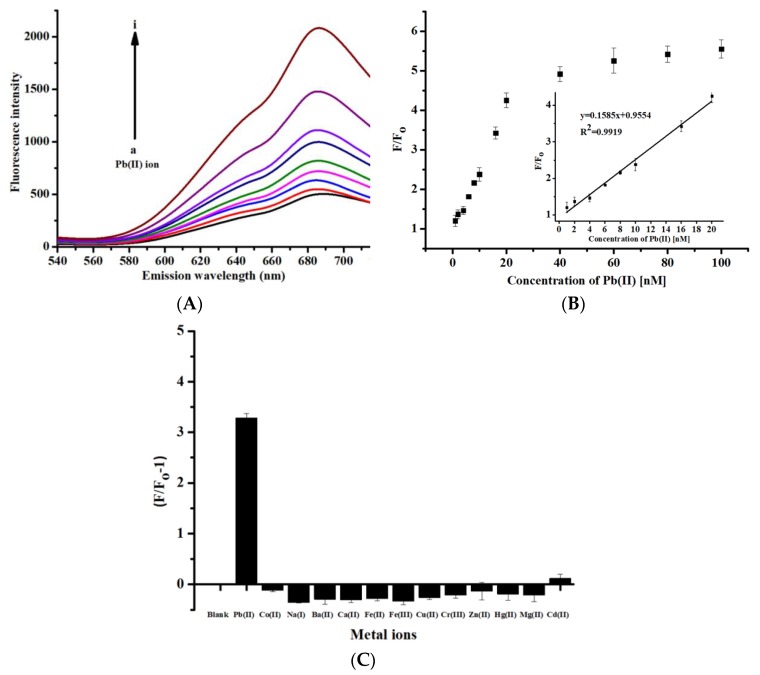
(**A**) Fluorescence spectra for the detection of Pb(II) ions by using *mw*_G-BSA-AuNCs (concentration of Pb(II): (**a**) 0 nM, (**b**)1 nM, (**c**) 2 nM, (**d**) 4 nM, (**e**) 6 nM, (**f**) 8 nM, (**g**) 10 nM, (**h**) 16 nM, and (**i**) 20 nM); (**B**) calibration curve for Pb(II) detection by using *mw*_G-BSA-AuNCs; (**C**) fluorescence response of *mw*_G-BSA-AuNCs for a 20 nM concentration of common metal ions (*n* = 5).

Figure 4A depicts the linear increase in the emission intensity of the probe at 685 nm under excitation with a wavelength of 370 nm as the concentrations of the Pb(II) ions increase from 1 to 20 nM after the incubation of the sensing platform for 6 min. Figure 4B illustrates the calibration (working) curves of *mw*_G-BSA-AuNCs, which were established by plotting the measured enhanced ratio (*F*/*F_o_*) of fluorescence emission intensity versus the added concentrations of the Pb(II) ion under the same excitation and emission wavelengths of the probe. A linear fit was observed for Pb(II) ion concentrations from 1 to 20 nM, with a correlation coefficient of *R*^2^ = 0.9919.

The limit of detection (LOD) for Pb(II) ions was 0.28 nM. This value was calculated by considering a signal-to-noise ratio of 3. The enhancement in the fluorescence intensity may be due to the chelation of Pb(II) ions by GE functional moieties, such as polyphenolic and flavonoid compounds. The detection limit was lower than those reported from 2018 to 2019, as listed in Table 1 [12,20,21,22,23]. Figure 4C illustrates the selectivity of *mw*_G-BSA-AuNCs for the detection of Pb(II) ions versus the detection of other metal ions, such as Cu(II), Co(II), Mg(II), Hg(II), Ca(II), Mn(II), Na(I), Ba(II) Zn(II), Fe(II), Fe(III), Cr(III) and Cd(II), in the sensing platform. The fluorescence intensities of all the added metal ions except Cd(II) decreased. Only a negligible enhancement was observed in the fluorescence of Cd(II) ions. These results indicated that *mw*_G-BSA-AuNCs are highly selective for Pb(II) ions over other metal ions.

**Figure 5:**
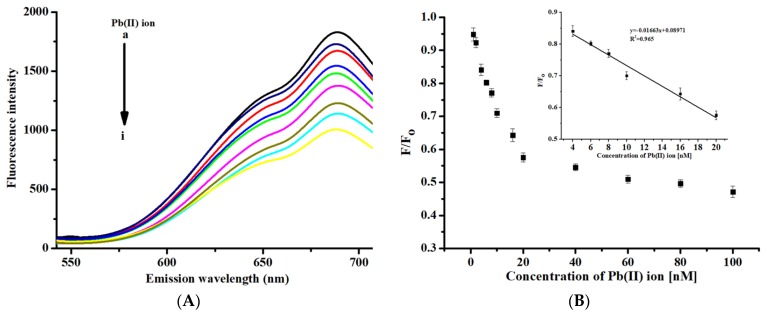

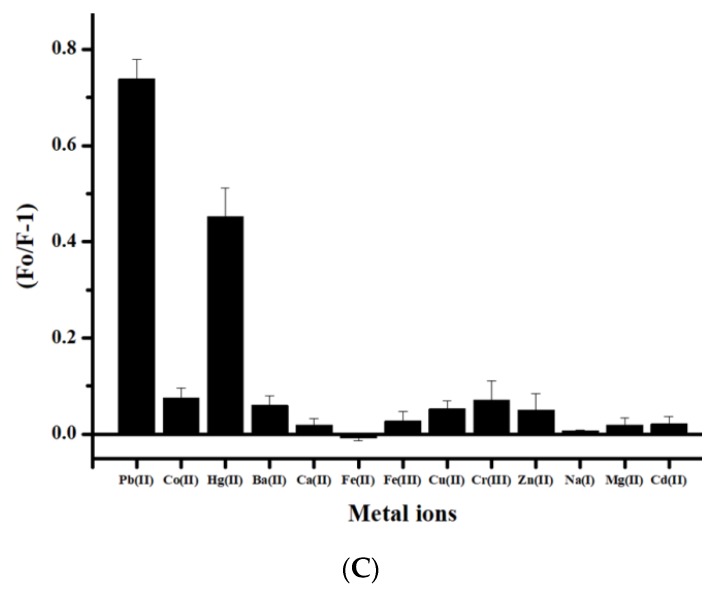
(**A**) Fluorescence spectra for the detection of Pb(II) ions by using *mw*_BSA-AuNCs (concentration of Pb(II): (**a**) 0, (**b**) 1, (**c**) 2, (**d**) 4, (**e**) 6, (**f**) 8, (**g**) 10, (**h**) 16, and (**i**) 20 nM); (**B**) calibration curve for Pb(II) detection by using *mw*_BSA-AuNCs; (**C**) fluorescence response of *mw*_BSA-AuNCs for a 20 nM concentration of metal ions (*n* = 5).

We also used the aforementioned procedure for detecting Pb(II) ions by using *mw*_BSA-AuNCs. A commonly used “quenching (off) method” was used as a reference for the proposed method. A decrease in the fluorescence emission intensity was observed at 689 nm (excitation wavelength: 370 nm) when introducing different concentrations of Pb(II) ions, as displayed in Figure 5A. This decrease may have been caused by the binding between the Pb(II) ions and the free functional groups of BSA. Figure 5B illustrates the corresponding calibration curve. A linear fit was observed for Pb(II) ion concentrations from 4 to 20 nM (*R*^2^ = 0.965), and the limit of detection was 1.1 nM for Pb(II) ion detection. Figure 5C depicts the results for the selectivity test of the *mw*_BSA-AuNCs when using the same metal ions [Co(II), Hg(II), Ba(II), Ca(II), Fe(II), Fe(III), Cu(II), Mn(II), Cr(III), Zn(II), Na(I), Mg(II), and Cd(II)] as in our GE-modified probe. The results indicated some interferences in the cases of Hg(II), Co(II), Cr(II), Zn(II), Cu(II) and Ba(II). This observation may be ascribed to the metallic interaction between Hg(II) and Au atoms, which diminishes the fluorescence intensity of *mw*_BSA-AuNCs. Overall, the “off-on” method of *mw*_G-BSA-AuNCs was superior to the “off” method of *mw*_BSA-AuNCs in terms of sensitivity and selectivity.

Table 1 compares the proposed method with methods reported in the literature from 2018 to 2019. The preparation time was dramatically reduced from several hours to 10 min. The detection limit was also many times lower than those provided by the previously proposed methods. The proposed method based on *mw*_G-BSA-AuNCs has clear advantages in terms of a rapid preparation time (min vs. h), lower detection limit, and its good selectivity. 

### 3.3. Detection of Pb(II) in Tap Water by Using mw_G-BSA-AuNCs Probes

The proposed method was used to measure the concentration of Pb(II) ions in tap water in order to exhibit its possible practical applications. Some known concentrations of Pb(II) ions were spiked into the tap water and incubated for 6 min. The fluorescence intensities were then measured at 685 nm for *mw*_G-BSA-AuNCs and at 689 nm for *mw*_BSA-AuNCs as a reference, for the same excitation wavelength of 370 nm. As presented in Table S1, an average recovery of 97.1% (*n* = 5) was observed for *mw*_G-BSA-AuNCs, and 89.9% was observed for *mw*_ BSA-AuNCs (*n* = 5). These results indicated that the *mw*_G-BSA-AuNC probe had a higher recovery than the *mw*_BSA-AuNC probe. Therefore, the new *mw*_G-BSA-AuNC probe is more feasible than the *mw*_BSA-AuNC probe for detecting Pb(II) ions in real samples.

## 4. Conclusions

Novel BSA-gold nanoclusters (*mw*_G-BSA-AuNCs) with garlic extract modifications were prepared through microwave-assisted rapid syntheses. The modified nanoclusters were characterized and used for the simple and sensitive detection of Pb^2+^ ions. The preparation time for the modified nanoclusters was 10 min, and the detection of Pb^2+^ ions required 6 min. The nanoclusters were stable, and their fluorescent intensities changed by less than 10% over 60 days. The detection limits and linear range for the “off-on” method of *mw*_G-BSA-AuNCs for Pb^2+^ ion detection were 0.28 nM and 1–20 nM, respectively. The recovery values of the *mw*_G-BSA-AuNCs probe for the detection of Pb(II) ions in tap water ranged from 93.8% to 102.2%, with an average value of 97.1%. The “off-on” method of *mw*_G-BSA-AuNCs can provide a lower detection limit, higher selectivity, and better recovery than the commonly used “off” method of *mw*_BSA-AuNCs for Pb^2+^ ion detection. The proposed method based on *mw*_G-BSA-AuNCs can be completed in a short time and can provide a lower detection limit than the methods previously proposed in the literature from 2018 to 2019. The microwave-assisted novel compound, *mw*_G-BSA-AuNCs, can be rapidly synthesized in a green manner and can provide a low detection limit, good selectivity, and a simple and fast reaction for Pb^2+^ ion detection.

## Figures and Tables

**Table 1:** Literature summary for the detection of the Pb(II) ion from some modifications of AuNCs in the last two years.

Probe	Reaction Time	Linear Range	Limit of Detection (LOD) (nM)	Year and Reference
GSH-AuNCs	24–96 h (35 °C)	10–190 nM	10	2018 [17]
PRT-AuNCs	12–24 h (35 °C)	80 nM–15 μM	24	2018 [18]
N-CD/GSH-AuNCs	24 h (70 °C)	2–60 μM	500	2019 [19]
DTT-BSA-AuNCs	1 h (50 °C)	4.8 nM–48 µM	1.3	2018 [10]
Graphene quantum dots (GQDs)GQDs/AuNP	16 h (110 °C)	50 nM–4 µM	16.7	2018 [20]
*mw*_G-BSA-AuNCs	10 min (70 °C)	1–20 nM	0.28	This study

GSH: glutathione, PRT: protamine, DTT: dithiothreitol, N-CD: Nitrogen-doped carbon dot, GQDs: Graphene quantum dots, AuNP: Gold nanoparticle.

## Supplementary Materials

The following are available online at https://www.mdpi.com/2079-4991/10/1/94/s1, Table S1 Detection of Pb(II) ions in spiked tap water by using *mw*_G-BSA-AuNCs, Figure S1 TEM image of synthesized (a) *mw*_G-BSA-AuNCs and (b) *mw*_BSA-AuNCs, Figure S2 Optimization of the fluorescence emission intensity of *mw*_G-BSA-AuNCs for different parameters. (a) reaction temperature (b) reaction time (c) GE concentration and (d) pH effect, Figure S3 Fluorescence spectra of *mw*_BSA-AuNCs for different reaction temperatures. Figure S4 Optimization of the fluorescence emission intensity *mw*_G-BSA-AuNCs of for different incubation times.
