# Supplementary material for: Microwave Synthesis of Gold Nanoclusters with Garlic Extract Modifications for the Simple and Sensitive Detection of Lead Ions
Source: Nanomaterials (Basel). 2020 Jan 2;10(1):94. doi: 10.3390/nano10010094 (PMC7023150; doi:10.3390/nano10010094)
Supplement: Supplementary file 1 [file nanomaterials-10-00094-s001.pdf]

Supplementary materials

Table S1. Detection of Pb(II) ions in spiked tap water by using *mw*_G-BSA-AuNCs.

Probe	Added Pb(II), nM	Detected Pb(II), nM (± SD)	Recovery (%)
*mw*_G-BSA-AuNCs	3	2.81 (± 0.15)	93.81
	5	4.91 (± 0.12)	98.33
	9	8.47 (± 0.18)	94.16
	15	15.33 (± 0.10)	102.2
*mw*_BSA-AuNCs	5	4.79 (± 0.74)	95.94
	9	7.33 (± 0.76)	81.44
	15	13.86 (± 0.63)	92.42

(n=5)

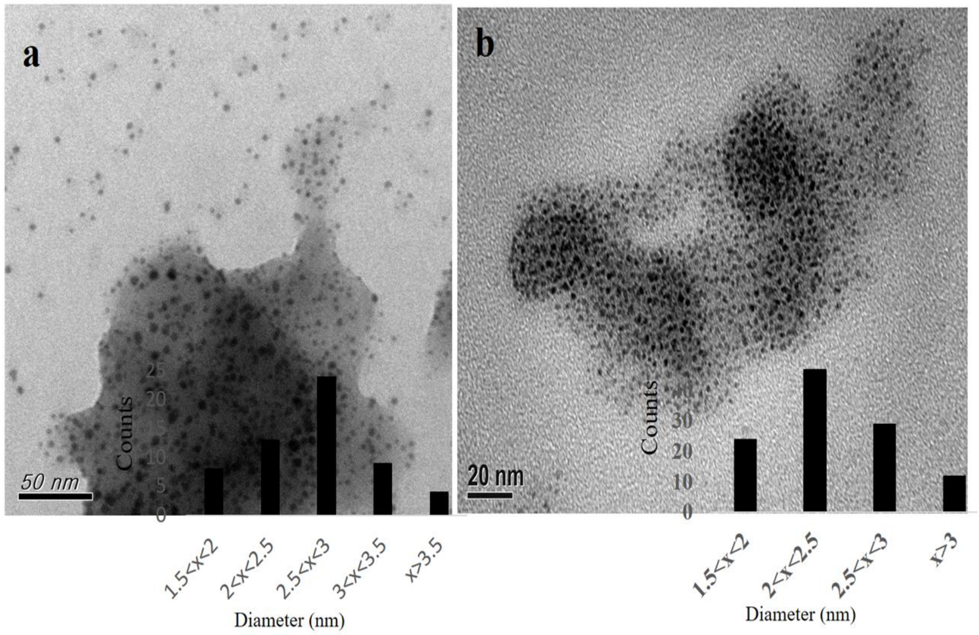

Figure S1. TEM image of synthesized (a) *mw*_G-BSA-AuNCs and (b) *mw*_BSA-AuNCs.

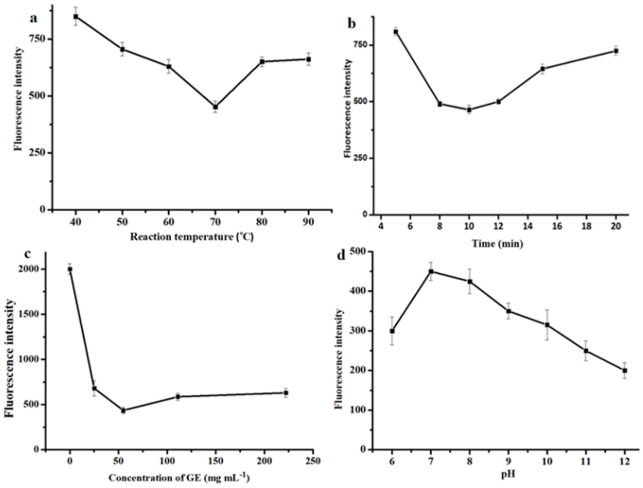

Figure S2. Optimization of the fluorescence emission intensity of *mw*_G-BSA-AuNCs for different parameters. (**a**) reaction temperature (**b**) reaction time (**c**) GE concentration and (**d**) pH effect.

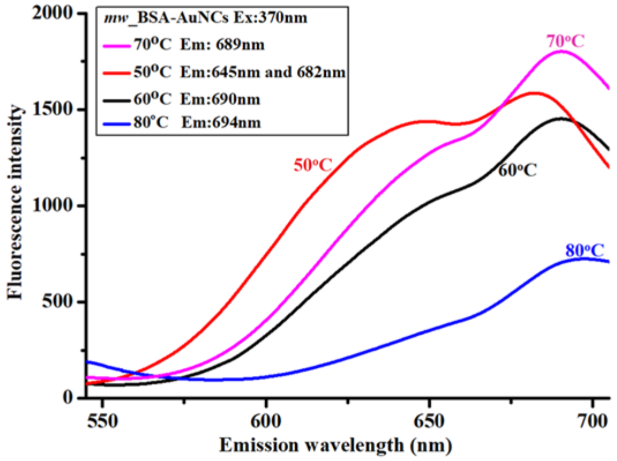

Figure S3. Fluorescence spectra of *mw*_BSA-AuNCs for different reaction temperatures.

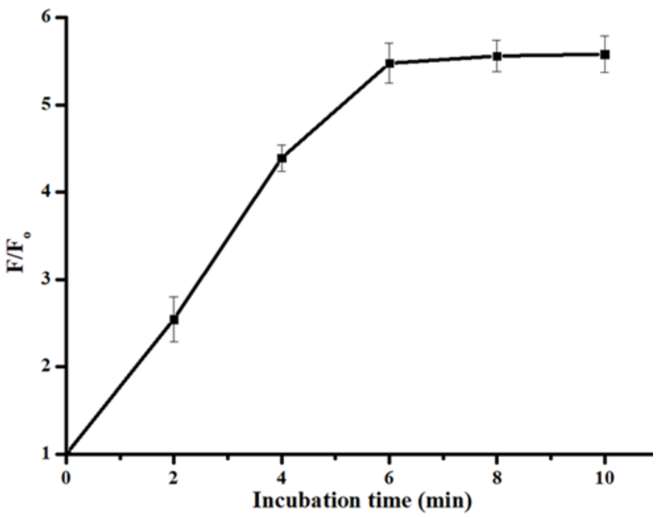

Figure S4. Optimization of the fluorescence emission intensity *mw*_G-BSA-AuNCs of for different incubation times.
